# Prior Hydrologic Disturbance Affects Competition between Aedes Mosquitoes via Changes in Leaf Litter

**DOI:** 10.1371/journal.pone.0128956

**Published:** 2015-06-02

**Authors:** Cassandra D. Smith, T. Zachary Freed, Paul T. Leisnham

**Affiliations:** Ecosystem Health and Natural Resource Management, Department of Environmental Science and Technology, University of Maryland, College Park, Maryland, United States of America; University of Missouri, UNITED STATES

## Abstract

Allochthonous leaf litter is often the main resource base for invertebrate communities in ephemeral water-filled containers, and detritus quality can be affected by hydrologic conditions. The invasive mosquito *Aedes albopictus* utilizes container habitats for its development where it competes as larvae for detritus and associated microorganisms with the native *Aedes triseriatus*. Different hydrologic conditions that containers are exposed to prior to mosquito utilization affect litter decay and associated water quality. We tested the hypothesis that larval competition between *A*. *albopictus* and *A*. *triseriatus* would be differentially affected by prior hydrologic conditions. Experimental microcosms provisioned with *Quercus alba* L. litter were subjected to one of three different hydrologic treatments prior to the addition of water and mosquito larvae: dry, flooded, and a wet/dry cycle. Interspecific competition between *A*. *albopictus* and *A*. *triseriatus* was mediated by hydrologic treatment, and was strongest in the dry treatment vs. the flooded or wet/dry treatments. *Aedes triseriatus* estimated rate of population change (*λ'*) was lowest in the dry treatment. *Aedes albopictus λ'* was unaffected by hydrologic treatment, and was on average always increasing (i.e., > 1). *Aedes triseriatus λ'* was affected by the interaction of hydrologic treatment with interspecific competition, and was on average declining (i.e., < 1.0), at the highest interspecific densities in the dry treatment. Dry treatment litter had the slowest decay rate and leached the highest concentration of tannin-lignin, but supported more total bacteria than the other treatments. These results suggest that dry conditions negatively impact *A*. *triseriatus* population performance and may result in the competitive exclusion of *A*. *triseriatus* by *A*. *albopictus*, possibly by reducing microbial taxa that *Aedes* species browse. Changing rainfall patterns with climate change are likely to affect competition between *A*. *triseriatus* and *A*. *albopictus*, probably enhancing negative competitive effects of *A*. *albopictus* on *A*. *triseriatus* in areas that experience drought.

## Introduction

Environmental variability inherently structures biological communities and is frequently observed in aquatic systems as hydrologic disturbances [1]. Flooding and drying of ephemeral habitats can affect aquatic invertebrate communities via direct mortality (e.g., [2–3]). Direct mortality and the resultant changes to the composition of communities from hydrologic changes have been relatively well-studied in ponds (e.g., [4]), streams (e.g., [1]), and some ephemeral container systems, such as water-filled tree holes (e.g., [5]). Hydrologic disturbances can also indirectly affect aquatic invertebrate communities by altering the quantity and quality of their food resources (e.g., detritus, microorganisms) or by changing solute concentrations [6]. These indirect effects can alter the outcome of interspecific competition, which is a strong influence on community structure under resource-limited conditions in ephemeral habitats (e.g., [5]). However, it is still unclear whether hydrologic disturbances intensify or relax interspecific competition via changes in resource availability and overall habitat quality.

Inundation of aquatic habitats can increase the decomposition rate of detritus, the availability of nutrients, and the growth of microorganisms that facilitate further detritus decomposition and provide the majority of food for many detrivorous invertebrates [7]. Increased detritus decomposition and the growth of associated edible microorganisms are expected to relax interspecific resource competition. Inundation in a container habitat is expected to also dilute soluble toxins that leach from litter (e.g., phenols, tannins, lignin), and that can negatively affect invertebrate survival and development (e.g., [8]). If a habitat floods completely and overflows, resources and toxins may be removed from the system, which could intensify competition for food resources or decrease the direct impacts of toxins. Habitat drying would likely also increase the concentration of toxins, which could reduce microbial growth and increase resource competition. Increasing foliar toxins could therefore affect the outcome of interspecific competition if species are differentially susceptible to the direct effects of toxins or reduced food resources (e.g., [9–10]). It is unclear how complete habitat drying may affect concentrations of toxins or the quality and quantity of edible microorganisms after habitats are re-inundated. Therefore, we are still uncertain of the effects of habitat flooding and drying on the outcome of competitive interactions in aquatic communities.

Water-filled natural (e.g., treeholes, plant axils) and human-made (e.g., bird baths, discarded tires, trash receptacles) containers are ephemeral aquatic habitats occupied by the developmental stages of invertebrates [11]. *Aedes* mosquitoes, and the aquatic containers they inhabit, are model systems in which to study changes in habitat quality and interspecific competition [12]. Allochthonous leaf litter commonly forms the energetic basis of these mosquito communities [13], and mosquito larvae feed upon associated microorganisms [14]. The hydrologic regime of containers may affect the decay rate of litter, the release of nutrients, the release of tannins, or any combination of these processes. Tannins are toxic to mosquito larvae at high concentrations (e.g., [15–17]), and are likely to negatively affect microbial growth [18].

The Asian tiger mosquito, *A*. *albopictus* (Skuse 1894), invaded the continental United States in the mid-1980s [19]. Since then, it has spread throughout the eastern United States and become one of the most common human-biting mosquitoes in its new range [19]. *Aedes albopictus* utilizes natural and human-made containers where it co-occurs with the native congener the eastern treehole mosquito, *A*. *triseriatus* (Say 1823). *Aedes albopictus* is a competent vector for West Nile virus, dengue virus, eastern equine encephalitis virus, and La Crosse encephalitis virus [20–22]. *Aedes triseriatus* is a vector for La Crosse encephalitis virus [23]. Past laboratory and field studies have consistently shown that *A*. *triseriatus* is an inferior resource competitor to *A*. *albopictus* [24–26], and that interspecific competition almost certainly controls mosquito community structure in container habitats (see review by Juliano [12], and references therein). Despite its competitive inferiority, *A*. *triseriatus* has persisted in many areas within the range of *A*. *albopictus* [27].

Multiple studies have shown that container hydrology can affect the development of *A*. *albopictus* and *A*. *triseriatus*. Juliano and Stoffregen showed that *A*. *triseriatus* larvae pupated earlier and at smaller sizes in containers with decreasing water volumes than in containers with constant volumes [28]. Alto and Juliano showed that containers with decreasing water volume produced larger emerging adult *A*. *albopictus* than in containers that were continuously wet habitats [29]. An experiment by Aspbury and Juliano that allowed some containers to dry completely before re-inundation showed that *A*. *triseriatus* species had a lower estimated rate of population increase, slower larval development, and lower adult mass in such containers compared to in containers that were always wet [6]. Although these studies demonstrate the importance of container hydrology on the population ecology of both *A*. *triseriatus* and *A*. *albopictus*, there have been no studies to examine interspecific competition between these two, or any other, mosquito species along a hydrologic gradient within ephemeral container habitats.

Theoretical and empirical work indicates that with limiting food in a constant environment, interspecific resource competition should result in competitive exclusion [30]. However, competitors can escape local extinction via a number of mechanisms, including differential resource use (e.g., [30]) or trade-offs between competitive ability and environmental tolerances (e.g., [31]). Although the abiotic environment can affect the distributions of species across varying environments, it is the role of the abiotic environment in modifying biotic interactions such as competition that can have the most important effects on species distributions and co-occurrence. Interspecific differences in utilizing food resources or tolerating foliar tannins that vary with hydrologic disturbances may reduce, or even reverse, the competitive superiority of *A*. *albopictus* over *A*. *triseriatus* when these species utilize container habitats, but this has yet to be rigorously tested [12].

The goal of this study is to use *A*. *triseriatus* and *A*. *albopictus* to test the hypothesis that hydrologic regime prior to the colonization of mosquitoes affects interspecific competition among larvae by altering the quality of the food resources and the concentration of inhibitory toxins. Prior studies on mosquitoes have mostly examined the effects of hydrologic regimes on larval mosquito ecology in habitats while mosquitoes are utilizing them [28, 29]. However, many ephemeral habitats undergo hydrologic changes before mosquitoes colonize them, which may affect microbial food resources and toxins in the environment. Aspbury and Juliano is one of the few studies that have tested the effects of prior hydrologic conditions on the ecology of a single species through changes in leaf litter quality [6]. However, they did not test effects of hydrologic conditions on co-occurring competing species within the same container that the hydrologic treatments were applied. Specific hypotheses that we tested in this study are: (1) that different hydrologic regimes (dry, flooded, wet/dry) will affect leaf decay rates, tannin concentrations, and bacterial abundance, and (2) that subsequent competition between *A*. *triseriatus* and *A*. *albopictus* will be mediated by these prior hydrologic regimes.

## Materials and Methods

Mosquitoes in the competition experiment were F2-F3 generation individuals of laboratory colonies, which originated from collections of disused trash containers and tree-holes in Maryland, USA. All mosquito collections were on public lands and no permission was required. Field collections (various sites: latitude. 38.9–39.3, longitude. -76.6–76.9) did not involve endangered or protected species. Field collected larvae were reared to adulthood at 26°C at 16:8 (L:D) h photoperiod and then released into 0.5-m3 cages. Adults were kept at 26°C and 75% RH at 16:8 (L:D) h photoperiod. Adults had continuous access to 20% sugar solution. Females were regularly fed anesthetized mice and laid eggs on seed paper in water-filled cups. IACUC approval was granted by University of Maryland R-12-41.

### Pre-competition hydrologic treatment

Habitat microcosms (400 mL plastic cups) were treated to emulate one of three broad hydrologic treatments prior to mosquito colonization: dry, flooded, and wet/dry. Each cup was provisioned with 0.5 g senescent dried white oak (*Quercus alba* L.) leaf litter cut into approximately 2.5 cm^2^ pieces. Flooded treatment cups were filled with and maintained at 140 mL of distilled water. Wet/dry cups were also initially filled with 140 mL of distilled water but were allowed to dry completely until no dampness was detected in the cup or on the leaves after gentle shaking and visual inspection. Dry cups were not filled with water. All cups were placed in an environmental chamber set at 14:10 (L:D) photoperiod and 26°C. Cups were rotated weekly to reduce potential chamber effects.

After 43 days, when the water in all wet/dry cups had evaporated completely, 250 mL of distilled water containing 100 μL of pond water inoculum was added to all cups from each treatment and mixed. A total of 140 mL was poured out of each flooded treatment cup to mimic overflow so that each cup contained 250 mL, similar to each cup in the dry and wet/dry treatments. Coarse detritus pieces stuck to the bottom of cups and were retained. However, it is likely that some fine detritus material suspended in the water columns of flooded cups was lost when water was emptied.

### Mosquito Competition Experiment

Competition trials were initiated five days after the cups were re-filled with water using 10 density combinations of *A*. *albopictus* and *A*. *triseriatus* (*A*. *albopictus*: *A*. *triseriatus*: 10:0, 20:0, 40:0, 10:10, 10:30, 20:20, 30:10, 0:40, 0:20, 0:10). Thirty combinations of density x habitat treatment were established with three replicates of each combination (90 cups). *Aedes albopictus* and *A*. *triseriatus* eggs (F_2_-F_3_ generation individuals of laboratory colonies) were synchronously hatched in a 0.3 g/L lactalbumin solution. Within 24 hrs of hatching, larvae were rinsed and added to cups and the experiment began. Twenty-six days after mosquitoes were added, 0.5 grams of dry litter were added to mimic the natural addition of leaf material in the field.

Pupae were removed from cups daily and placed in separate vials until they eclosed as adults. After each pupa eclosed, its sex was determined, and the proportion of female survivorship, mean development time, and mean female body size (dry mass) were recorded for each species from each cup. These fitness variables were used to estimate the finite rate of population change for each species (*λ’*) [32]:

λ'=exp[ln[(1/N0)∑xAxf(wx)]D+[∑xxAxf(wx)∑xAxf(wx)]]

where N_0_ is the initial number of females (assumed to be 50% per cup [33]), *x* is the mean development time (measured in days), *A*_*x*_ is the mean number of females eclosing on *x*, and *w*_*x*_ is the mean wing length (millimeters) on *x*. The function *f*(*w*_*x*_) is different for each mosquito species and describes the fecundity and female wing length relationship. The function for *A*. *albopictus* was *f(w*_*x*_ *)* = -121.240+78.02*w*_*x*_ [34]. The function for *A*. *triseriatus* was *f(w*_*x*_ *)* = (1/2) exp[4.5801 + 0.8926(ln*w*_*x*_)] − 1 [35]. D is the mean days it takes for an adult mosquito to mate, bloodfeed, and oviposit, and is estimated at 14 days for *A*. *albopictus* [34] and 12 days for *A*. *triseriatus* [35]. The mosquito competition experiment extended until 51 days, when all individual mosquitoes had either eclosed or died.

### Water Quality Experiment

An additional 36 cups (12 of each litter treatment) with no mosquitoes were established to measure baseline water quality. Hydrologic treatments were applied in exactly the same way as in the mosquito competition experiment, with water in the wet/dry cups taking 45 days to until no dampness was detected in the cup or on the leaves after gentle shaking and visual inspection. After the wet/dry treatment cups had evaporated completely, 250 mL of distilled water containing 100 μL of pond water inoculum were added to all cups from each treatment. Six cups were randomly selected from each treatment (totaling 18 cups) and destructively sampled 9 and 13 days after being re-inundated. To give a standardized representation of tannin-lignin concentrations and bacteria abundances among litter treatments, each cup had its water homogenized and then two 20 mL samples were pipetted from the water column. Tannin-lignin concentrations were measured from one water sample of each cup, using a Hach calorimeter and TA-3 Test Kit (Hach Company, Loveland CO). The second water sample of each cup was used for serial dilution and plating on general microbiological media (Tryptone yeast extract [TYE] agar, Sigma-Aldrich Co., St. Louis, MO) to determine the relative abundance of fast-growing, aerobic, heterotrophic bacteria. Remaining litter was removed from each cup by using a 105 μm sieve. Litter was dried (>48 h at 50°C) and weighed to the nearest 0.1 g, and dry mass was recorded to calculate litter decay rate.

### Statistical Analyses

Two-way analyses of variance (ANOVA; PROC GLM, SAS Institute 2004) were used to test effects of hydrologic pre-treatment (dry, flooded, wet/dry) and day on proportion of litter decay, bacterial abundance, and tannin-lignin concentration. A second set of two-way ANOVAs were used to test effects of hydrologic pre-treatment (dry, flooded, wet/dry), mosquito densities (continuous variables), and their interactions on survivorship to adulthood, mean time to eclosion, mean body size, and *λ'* of each species. To compensate for a potential increase in experimentwise type I error rate due to running linear models for multiple dependent variables on the same experimental units, we used a sequential Bonferroni adjustment for tests for each model, with experimentwise α = 0.05. However, we note that there is no consensus in the literature on whether or not to apply corrections for multiple tests or how to apply corrections (e.g., [36–37]); thus, we report all *P* values so that the reader can interpret them as they are. Post-hoc pairwise mean comparison tests with Bonferroni corrections were performed on all significant main effect and interaction treatments. Error degrees of freedom (df) differed if no female mosquitoes emerged from the microcosms. To meet the assumptions of normality, we log_10_ transformed the bacterial abundance and arcsine square-root transformed survivorship. Litter decay rate did not meet the assumption of normality and could not be remedied by transformations, therefore we tested for effects using randomization ANOVA (Randomization wrapper for SAS PROCs) [38].

## Results

### Water Quality Experiment

**Fig 1:**
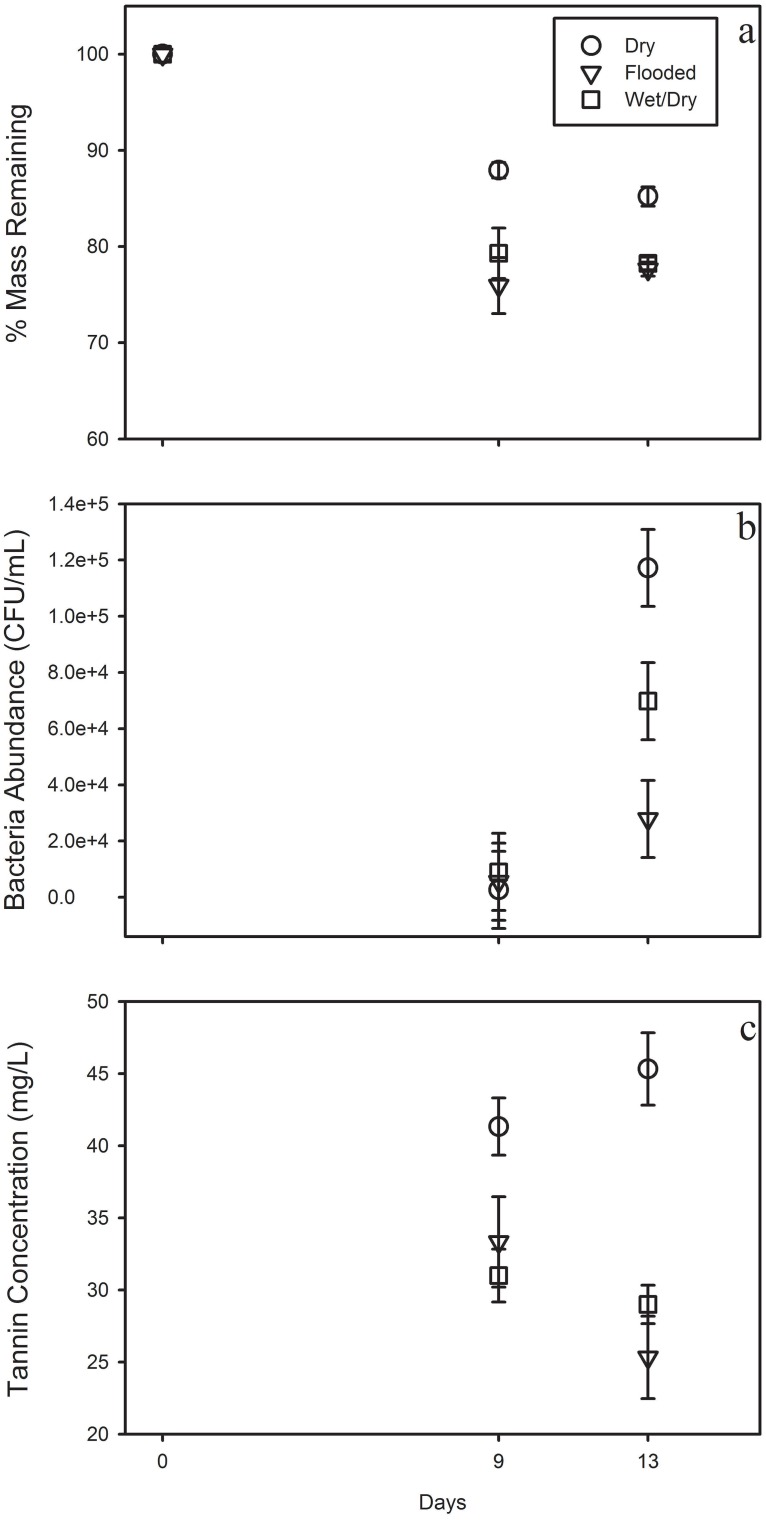
Water Quality Experiment Results. *Quercus alba* leaf litter (0.5 grams) was subjected to three hydrologic regimes: previously dried leaves, flooded leaves, and wet/dry leaves. Figures show differences among leaf litter in decay rate (a), bacterial abundance (b), and tannin concentration (c) as measured 9 and 13 days after inundation.

Leaf litter decay rate was affected by hydrologic treatment, day, and hydrologic treatment × day interaction (Table 1 and S1 Dataset). Litter in the dry hydrologic treatment decayed more slowly than litter in the flooded and wet/dry treatments (Fig 1a). Bacterial abundance was affected by days and hydrology × day interaction (Table 1). Bacterial abundance did not vary among treatments until 13 days after inundation, with higher mean bacterial abundance in the dry treatment than in the flooded treatment (Fig 1b). Tannin-lignin concentration was affected by hydrology (Table 1), with litter in the dry treatment leaching more tannins than litter in the flooded and wet/dry treatments (Fig 1c).

**Table 1:** Randomization ANOVA results for proportion of litter decay and parametric ANOVA results for log_10_ transformed bacterial abundance (colony forming units, CFU/ml) and tannin-lignin concentration (mg/L) in cups exposed to one of three hydrologic treatments (dry, flooded, wet/dry).

	Proportion of litter decay	Bacteria			Tannin-lignin		
Source	*P*	*df*	*F*	*P*	*df*	*F*	*P*
Hydrologic treatment	**0.0020**	2	3.12	0.0589	2	22.4	**<0.0001**
Day	**<0.0001**	1	84.9	**<0.0001**	1	1.08	0.3073
Hydrologic treatment X Day	**0.0170**	2	6.39	**0.0049**	2	3.24	0.0534
Error		30			30		

Proportion of litter decay was measured 0, 9, and 13 days after inundation. Bacterial abundance and tannin-lignin concentration were measured 9 and 13 days after inundation. Effects significant at experimentwise α = 0.05 (sequential Bonferroni) are shown in bold.

### Mosquito Competition Experiment

**Fig 2:**
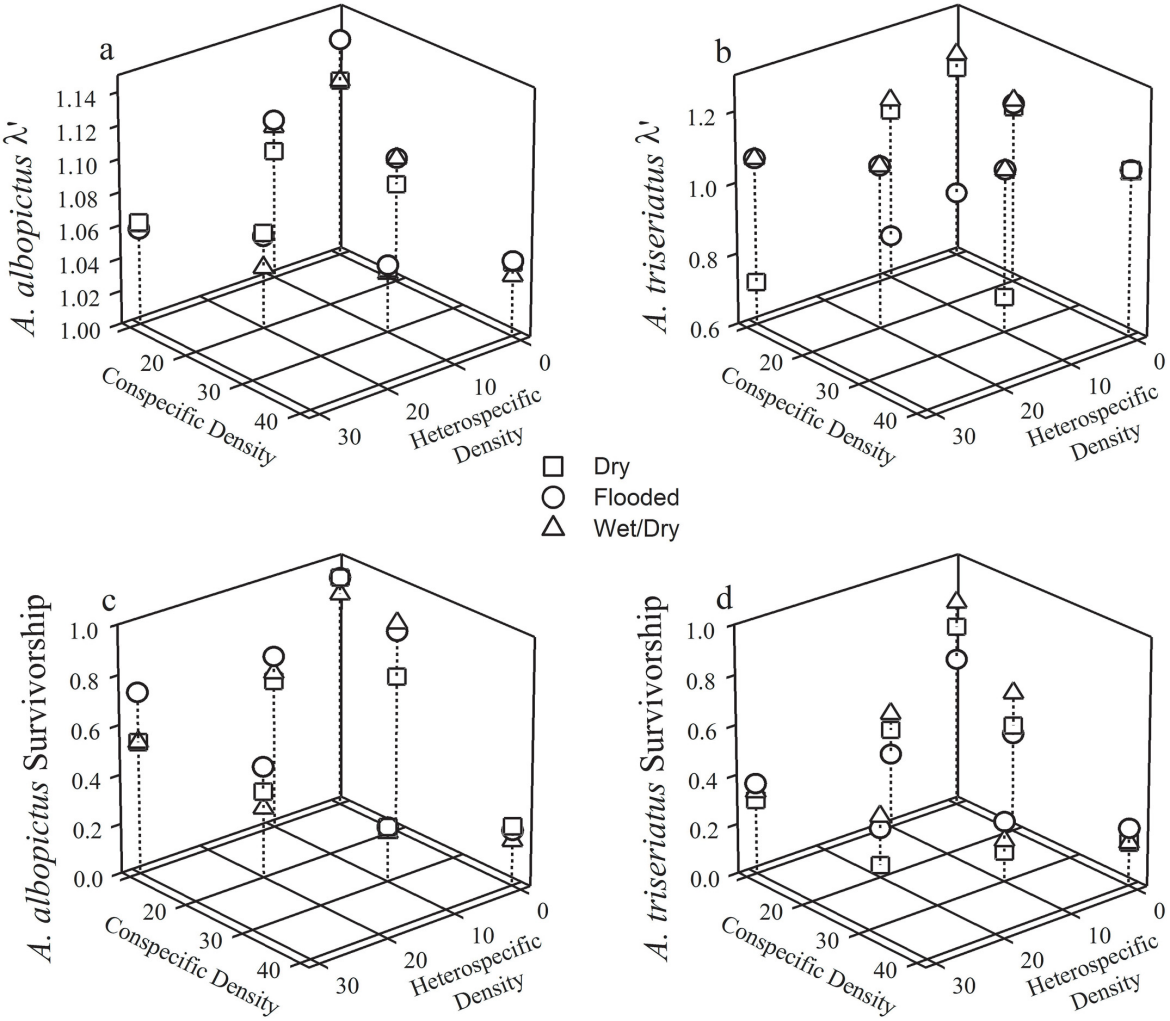
Mosquito Competition Results. Effects of competition densities and leaf litter on the finite rate of population increase *λ'* (a, b) and survivorship (b, c) of *Aedes albopictus* and *Aedes triseriatus*.

*Aedes albopictus λ'* was negatively affected by increasing heterospecific and conspecific densities (Table 2 and Fig 2a and S2 Dataset). However, there was no interaction between hydrologic treatment with either heterospecific or conspecific densities, indicating that the effects of competition were consistent among litter treatments. In contrast, *A*. *triseriatus λ'* was affected by an interaction of heterospecific (i.e., *A*. *albopictus*) density × hydrologic treatment (Table 2 and S2 Dataset). In the three high-density competition treatments (10:30, 30:10, and 20:20 densities) with the dry hydrologic pre-treatment, mean *Aedes triseriatus λ'* was < 1.0 when it co-occurred with *A*. *albopictus* (*λ'* = 0.72, 0.70, and 0.0, respectively) (Fig 2b). No *A*. *triseriatus* females emerged from any of the 20:20 cups in the dry treatment (Fig 2b). Mean *Aedes triseriatus λ'* was not negatively affected by heterospecific or conspecific densities in wet/dry and flooded treatments (regression slope coefficients = 0.00–0.01; P-values = 0.1151–0.6217), and mean *A*. *triseriatus λ'* was > 1.0 at all high (10:30, 30:10, 0:40, and 20:20) larval densities in these hydrologic treatments (Fig 2a). Mean *A*. *albopictus λ'* was > 1.0 regardless of hydrologic treatment and mosquito density, and higher or similar than *A*. *triseriatus λ'* among almost all treatment-density combinations. Increasing heterospecific and conspecific densities decreased both *A*. *triseriatus* and *A*. *albopictus* survivorship (Table 2 and Fig 2c and 2d). Increasing densities of heterospecifics and conspecifics increased the development time of *A*. *albopictus* and *A*. *triseriatus* females (Table 2). *Aedes albopictus* and *A*. *triseriatus* female mass was not affected by hydrologic treatment or increasing densities of mosquito larvae (Table 2).

**Table 2:** ANOVA results testing effects of mosquito densities and hydrologic treatment on *λ'*, survivorship, female mass, and female development time of *A*. *albopictus* and *A*. *triseriatus* in habitats exposed to one of three hydrologic treatments (dry, flooded, wet/dry).

	λ’			Survivorship			Female Mass			Female Development Time		
	*df*	*F*	*P*	*df*	*F*	*P*	*df*	*F*	*P*	*df*	*F*	*P*
*Aedes albopictus*												
Conspecific density	1	159	**<0.0001**	1	125	**<0.0001**	1	0.20	0.6549	1	128	**<0.0001**
Heterospecific density	1	75.2	**<0.0001**	1	32.6	**<0.0001**	1	2.45	0.1235	1	55.2	**<0.0001**
Hydrologic treatment	2	3.53	0.0364	2	2.62	0.0815	2	1.26	0.2911	2	1.69	0.1933
Conspecific X Hydrology	2	1.68	0.1961	2	0.96	0.3880	2	0.60	0.5509	2	0.76	0.4738
Heterospecific X Hydrology	2	2.08	0.1349	2	1.18	0.3148	2	0.90	0.4117	2	0.15	0.8600
Error	54			56			54			54		
*Aedes triseriatus*												
Conspecific density	1	0.16	0.6881	1	50.2	**<0.0001**	1	0.05	0.8165	1	63.5	**<0.0001**
Heterospecific density	1	4.42	0.0403	1	31.2	**<0.0001**	1	0.34	0.5643	1	62.3	**<0.0001**
Litter treatment	2	3.45	0.0389	2	2.61	0.0825	2	0.18	0.8335	2	3.65	0.0335
Conspecific X Hydrology	2	2.88	0.0647	2	1.89	0.1612	2	0.02	0.9845	2	2.55	0.0891
Heterospecific X Hydrology	2	7.32	**0.0015**	2	1.48	0.2363	2	1.49	0.2369	2	0.77	0.4703
Error	47			56			47			47		

Effects significant at experimentwise α = 0.05 (sequential Bonferroni) are shown in bold.

## Discussion

Our experiment shows that the hydrologic conditions of water-filled containers before mosquito utilization can affect leaf litter resource quality, water quality, and competition between the invasive mosquito *A*. *albopictus* and the native congener *A*. *triseriatus*. Litter in cups that were previously dry decayed more slowly, leached higher tannin-lignin concentrations, and promoted higher microbial abundance than litter in cups that were constantly flooded or that had been exposed to a wet/dry cycle. These changes in resource and water quality were associated with intense competitive effects of *A*. *albopictus* on *A*. *triseriatus λ’* in the dry treatment and no effects of competition on *A*. *triseriatus λ’* in the flooded and wet/dry treatments. These results suggest that less inundation due to less frequent rainfall events is likely to increase the existing competitive advantage of *A*. *albopictus* over *A*. *triseriatus*, whereas more frequent rainfall events may relax any competitive effects of *A*. *albopictus* on *A*. *triseriatus*.

*Aedes triseriatus* is able to persist in many areas that have been invaded by the competitively superior *A*. *albopictus* despite frequently co-occurring in natural and artificial containers (see review by Juliano and Lounibos [19] and references therein). Changing patterns of rainfall as a result of climate change are likely to modify gradients of competitive intensity between *A*. *albopictus* and *A*. *triseriatus* via numerous ecological mechanisms, including altering the quantity and quality of food resources and moderating the concentrations of toxins. In our study, mean *A*. *triseriatus λ'* indicated a declining population (i.e. < 1.0) in cups with high *A*. *albopictus* densities that had been exposed to dry conditions. If climate change leads to more frequent habitat drying events, we may expect *A*. *albopictus* to be more likely to competitively exclude *A*. *triseriatus* in areas where the native species is currently persisting. On the other hand, more frequent rainfall events appear to relax competitive effects of *A*. *albopictus* on *A*. *triseriatus*, thus promoting species coexistence.

Higher concentrations of tannin-lignin and slower litter decay may be expected to suppress microbial growth and resource availability to mosquito larvae in container habitats. In our study, *A*. *triseriatus* experienced the most severe competitive effects from *A*. *albopictus* in the dry treatment, but dry treatment cups actually had the highest bacterial abundances. One reason for this result may be that the bacterial communities we quantified only partially represented the microbial fauna that is consumed by *Aedes* larvae. *Aedes* mosquitoes have been found to consume bacteria both in the water column and on the surfaces of leaves and containers [39]. Bacterial communities that colonize surfaces may consist of different taxa as those in the water column. Although we homogenized the water of each cup before taking water samples, it is possible that our sample did not gather a complete representation of microbial taxa that were consumed by larvae in the cups. At least one past study has found that the presence of mosquito larvae in container habitats is associated with increased bacterial abundance in water and decreased bacterial abundance on leaf surfaces [40]. These results suggest that *Aedes* larvae may preferentially feed on leaf surface bacteria or that leaf surface bacteria do not replenish as quickly as water column bacteria. In our study, the dry treatment had higher mean tannin concentrations, and tannin concentration has been shown to delay mosquito development and decrease larval survival (e.g., [15, 16]). If tannins directly affected *A*. *triseriatus* larvae, we would expect lower *A*. *triseriatus λ'* in the dry treatment across all mosquito densities. Instead, we observed lower *A*. *triseriatus λ'* with high *A*. *albopictus* density, suggesting that tannins may have suppressed edible microbial food resources and thus intensified competition on *A*. *triseriatus*. Further, a laboratory study by Smith et al. showed that tannic acid negatively affected *A*. *albopictus* and *A*. *triseriatus λ'* between 100 and 500 mg/L [17]. Tannin-lignin concentrations in this experiment never exceeded 50 mg/L, so they were unlikely to directly affect the mosquitoes but may have been high enough to suppress production of edible (but not overall) microorganisms.

In this study, the heterotrophic plate count method was used to provide a broad comparison of bacterial abundances among litter treatments. This approach is consistent with numerous other studies of mosquito ecology in container habitats which have used culture techniques to measure bacterial abundance (e.g., [17, 41]). The study here is among the first to test the effects of hydrologic conditions on the outcome of interspecific competition between two mosquito species. Further research should investigate changes in microbial communities with hydrologic changes, specifically comparing changes among specific microbial taxa both in the water column and on litter surfaces, and in the guts of larvae to identify fauna that are likely important to mosquito ecology. Studies are increasingly using molecular tools, such as real-time quantitative polymerase chain reaction (qPCR) (e.g., [41]) or next-generation sequencing (e.g., [42]) to identify and quantify bacterial taxa, and thus better understand the composition and structure of microbial communities and their relationships with mosquito larvae. Such tools need to be applied in future studies that directly compare containers under different hydrologic regimes.

*Aedes albopictus λ'* was negatively affected by densities of *A*. *triseriatus* and *A*. *albopictus* in a similar pattern among all three hydrologic treatments, indicating that prior hydrologic conditions did not mediate the effects of competition on *A*. *albopictus*. Inconsistent with our results, Aspbury and Juliano found *A*. *triseriatus λ'* to be negatively affected by litter in tree holes that had been subjected to conditions broadly similar to the wet/dry treatment in the study here [6]. Aspbury and Juliano utilized a much longer drying time of the leaves (70 days) prior to their re-inundation, which may have had different effects of drought on microbial communities than in our study, and caused different results [6]. In our study, mean *A*. *albopictus λ'* was > 1.0 regardless of hydrologic treatment and mosquito density, and higher or similar than *A*. *triseriatus λ'* among almost all treatment-density combinations. These results indicate that, overall, *A*. *albopictus* is the superior competitor for food resources and is less affected by prior hydrologic regimes than *A*. *triseriatus*.

Hydrologic disturbances, such as droughts and floods, have been shown to affect the structure and function of ecological communities [43, 44], as well as alter risks from vector-borne infectious diseases, including that of West Nile Virus [45]. Ecosystem effects of climate change may act through subtle but complex changes to interspecific interactions, such as competition, predation, and parasitism. This study provided evidence that competition between the invasive mosquito *A*. *albopictus* and the native *A*. *triseriatus* is affected by the hydrologic conditions of container habitats prior to mosquito colonization. The hydrologic effect of climate change on mosquito competition is likely to have consequences for the spread of invasive species and persistence of natives, and may ultimately alter disease risks to humans. Future studies should test mosquito competition along a gradient of other climatic variables (e.g., temperature, CO_2_) in addition to hydrologic scenarios to determine the effect of climate change on mosquito populations.

## Supporting Information

S1 Dataset Raw data of the water quality experiment. Provided in adherence to the PLOS policy to make all data underlying the findings described in this manuscript fully available.
(XLSX)

S2 Dataset Raw data of the mosquito competition experiment. Provided in adherence to the PLOS policy to make all data underlying the findings described in this manuscript fully available.
(XLSX)
