# Correction: Prior Hydrologic Disturbance Affects Competition between Aedes Mosquitoes via Changes in Leaf Litter

**DOI:** 10.1371/journal.pone.0152956

**Published:** 2016-03-30

**Authors:** Cassandra D. Smith, T. Zachary Freed, Paul T. Leisnham

The following information is missing from the Funding section: Funding for this work was provided by the NSF-Couple Natural Human Systems Program (DEB-1211797).
